# Photodynamic therapy improves the clinical efficacy of advanced colorectal cancer and recruits immune cells into the tumor immune microenvironment

**DOI:** 10.3389/fimmu.2022.1050421

**Published:** 2022-11-17

**Authors:** Baohong Gu, Bofang Wang, Xuemei Li, Zedong Feng, Chenhui Ma, Lei Gao, Yang Yu, Jing Zhang, Peng Zheng, Yunpeng Wang, Haiyuan Li, Tao Zhang, Hao Chen

**Affiliations:** ^1^ Second Clinical Medical College, Lanzhou University, Lanzhou, Gansu, China; ^2^ Department of Oncology, The First Hospital of Lanzhou University, Lanzhou, Gansu, China; ^3^ Key Laboratory of the Digestive System Tumors of Gansu Province, Lanzhou, Gansu, China; ^4^ Department of Oncology, Lanzhou University Second Hospital, Lanzhou, Gansu, China

**Keywords:** photodynamic therapy (PDT), colorectal cancer, clinical efficacy, tumor microenvironment, immunity

## Abstract

**Objective:**

Although photodynamic therapy (PDT) has been proven effective in various tumors, it has not been widely used as a routine treatment for colorectal cancer (CRC), and the characteristics of changes in the tumor microenvironment (TME) after PDT have not been fully elucidated. This study evaluated the efficacy of PDT in patients with advanced CRC and the changes in systemic and local immune function after PDT.

**Methods:**

Patients with stage III-IV CRC diagnosed in our hospital from November 2020 to July 2021 were retrospectively analyzed to compare the survival outcomes among each group. Subsequently, short-term efficacy, systemic and local immune function changes, and adverse reactions were assessed in CRC patients treated with PDT.

**Results:**

A total of 52 CRC patients were enrolled in this retrospective study from November 2020 to July 2021, and the follow-up period ended in March 2022. The overall survival (OS) of the PDT group was significantly longer than that of the non-PDT group (*p*=0.006). The objective response rate (ORR) and disease control rate two months after PDT were 44.4% and 88.9%, respectively. Differentiation degree (*p*=0.020) and necrosis (*p*=0.039) are two crucial factors affecting the short-term efficacy of PDT. The systemic immune function of stage III patients after PDT decreased, whereas that of stage IV patients increased. Local infiltration of various immune cells such as CD3^+^ T cells, CD4^+^ T cells, CD8^+^ T cells, CD20^+^ B cells and macrophages in the tumor tissue were significantly increased. No severe adverse reactions associated with PDT were observed.

**Conclusion:**

PDT is effective for CRC without significant side effects according to the available data. It alters the TME by recruiting immune cells into tumor tissues.

## 1 Introduction

Colorectal cancer (CRC) is one of the most common gastrointestinal malignancies. According to the global cancer statistics 2020, the overall incidence of CRC has jumped to third place, ranking second among all cancer deaths (1). In China, CRC is the second most common cancer and the fifth leading cause of cancer death, with about 560,000 new cases and 290,000 deaths. China has become the country with the most significant number of new cases and deaths of CRC every year (1, 2).

CRC has an insidious onset, and many patients are already at an advanced stage when the tumor is detected (3), losing the opportunity for radical surgical treatment. The 5-year relative survival rate for the localized lesion is higher than 90%, whereas that for the lesion with concomitant distant metastasis is less than 10% (4). However, even if they are eligible for surgery, patients with low rectal cancer usually prefer to remain anal to avoid quality-of-life changes associated with a colostomy or low anterior resection syndrome (5). Traditional CRC therapies such as chemotherapy and radiotherapy often accompany myelosuppression, non-targeting tumor-damaging normal cells, and multidrug resistance (6). Some patients may not be treated on time or give up halfway due to severe side effects and adverse events that cannot be tolerated. Additionally, cell therapy, immunotherapy, and targeted therapy have shown great potential in treating CRC, but the low benefit rate and high cost limit the number of people who benefit (6). These current situations pose severe challenges to the current treatment of CRC.

Photodynamic therapy (PDT) can effectively complement the abovementioned CRC treatment methods. Due to a series of advantages such as high safety, robust targeting, low invasiveness, and fewer side effects, PDT may even become one of the conventional anti-tumor treatments in the future (7). The principle of PDT is utilizing a specific wavelength of red light to irradiate and excite photosensitizers taken up by tumor tissue to generate cytotoxic factors such as reactive oxygen species, which induce tumor cell death (8). In addition to the direct killing effect, the anti-tumor effect of PDT is primarily based on its impact on the body’s immune and inflammatory responses. PDT frequently provokes a solid acute inflammatory reaction, which increases the immunogenicity of tumor cells and stimulates the body’s anti-tumor immune response (9).

Although numerous studies and clinical data suggest that PDT can affect immune responses, the effect of PDT on the immune system of patients with CRC has not been reported so far, and many questions remain unanswered regarding CRC. For example, what effect does PDT have on systemic and local immune function in CRC patients of different clinical stages? Is there a connection between the two? It is necessary and interesting to clarify these questions. Based on the evaluation of the safety and efficacy of PDT in the treatment of CRC, this study further explored the changes and relationship between the peripheral blood and tumor tissue local immune microenvironment of patients after PDT.

## 2 Materials and methods

### 2.1 Patient information

We retrospectively reviewed the data of all patients diagnosed with CRC from November 2020 to July 2021 in the Lanzhou University Second Hospital, Lanzhou, China. Selected patients were divided into the PDT group (n=8), PDT+systemic therapy (ST) group (n=10), ST group (n=19), and untreated group (n=15) according to what treatment the patient has received. The primary inclusion criteria were patients with a first diagnosis of CRC and not receiving any conventional anti-tumor therapy, clinical stage III to IV, refusal to undergo palliative surgery, or the presence of a physical adverse event that would make the surgery intolerable. All patient information was collected from medical records, including colonoscopy images, immunoassay data, and pathology reports. This study was approved by the Research Institutions Review Board and carried out under the ethical principles of the Declaration of Helsinki.

### 2.2 Photosensitizer and laser device

The photosensitizer was Hematoporphyrin (Milelonge Biopharmaceutical Co., LTD, China), molecular formula: C34H38N4O6, molecular weight: 598.70, which can be excited by a visible red light with a wavelength of 630 nm. The laser (Xingda Photoelectric Medical Instrument Co., LTD, China) emitted by the treatment light source is 630 nm with pulse output. The optical fiber is a columnar optical fiber, and the luminous band (Xingda Photoelectric Medical Instrument Co., LTD, China) at the end can be selected from 1 to 6 cm.

### 2.3 Therapeutic schedule

Patients were divided into the PDT group and the non-PDT group. Patients in the PDT group were given Hematoporphyrin 48 hours before treatment. Before infusion, the original solution was taken for a skin scratch test on the patient’s forearm. If there was no swelling or induration within 15 minutes, Hematoporphyrin was added to 250ml normal saline (NS) at 5mg/kg, and intravenous infusion was completed within one hour. The patient was subsequently protected from exposure to sunlight. PDT therapy was initiated 48h after infusion. An expandable uncovered metal intestinal stent (WallFlex, Boston Scientific) was placed before irradiation for patients with an obstruction or tumor that protruded significantly into the lumen to gain maximum treatment area. The appropriate columnar fiber was chosen according to the lesion size. If it is a circumferential growing tumor, the optical fiber is placed in the center of the lumen, and if an eccentric growing tumor, the optical fiber is placed on the tumor surface as much as possible, far away from the normal bowel wall. Segmented irradiation is adopted when the lesion exceeds the optical fiber’s length. Generally, the irradiation parameters are as follows: wavelength is 630 nm, power is 800nW, energy density is 200J/cm^2^, and continuous irradiation for 3 or 4 days. Necrotic tissue was removed by colonoscopy 48 hours after PDT. The patient will decide whether to combine with other tumor treatments one week later. Patients should avoid direct exposure to sunlight or any other strong light source for one month after the photosensitizer injection.

The non-PDT group did not receive PDT intervention, and the other treatments were the same as the PDT group.

### 2.4 Response evaluation of PDT

#### 2.4.1 Short-term response evaluation

The surface necrosis of the irradiation site was observed by colonoscopy 48h after PDT. The evaluation of the necrosis degree according to the following criteria: Mild: the necrosis area of the lesion is less than 30%; Moderate: 30%-70% necrosis area of lesion area; Severe: necrosis occurs over 70% of the lesion area.

After two months, patients who received PDT need to undergo computed tomography (CT) or magnetic resonance imaging (MRI) examinations to compared with the results before treatment. Patients should undergo colonoscopy again to determine the degree of intestinal tract patency and tumor necrosis. Afterward, experienced researchers record target lesion measurements, development of new lesions, and tumor response in patients treated with PDT according to the response assessment criteria in Response to Solid Tumors (RECIST) 1.1 (10). Changes in quality-of-life before and after treatment were assessed using the KPS score.

#### 2.4.2 Assessment of adverse reactions

Patients were recorded for fever, abdominal pain, intestinal perforation, bleeding at the end of PDT treatment, and photoallergic reactions under strict photoprotective conditions.

### 2.5 Evaluation of immune function

Peripheral blood was collected before and 48 hours after PDT. The systemic immune function status of CRC patients was assessed by flow cytometry. The antibodies used for flow cytometry, including CD3 (#Z6410026), CD19 (#Z6410014), CD127 (Z6410046) were purchased from QuantoBio Biotechnology (Beijing, China), antibodies against CD4 (#347413), CD8 (#348793), CD25 (#555434), CD45RO (#340438), CD45RA (#564359) and CD56 (#335791) were bought from BD Biosciences (San Jose, CA,USA). Immunohistochemistry (IHC) was used to detect local immune cell infiltration of the tumor before and 48h after PDT. The tissue materials were obtained from the paraffin-embedded tissue samples used for preoperative diagnosis and postoperative efficacy evaluation. IHC-relevant antibodies against CD3 (#Kit-0003), CD4 (#RMA-0620), CD8 (#RMA-0514), CD20 (Kit-0001), CD56 (MAB-0743), and CD68 (Kit-0026) were obtained from Maixin Biotechnology (Fuzhou, China).

### 2.6 Follow-up

Clinical follow-up for all patients ended on March 10, 2022. The medical history data were obtained from regular inspection records, and the survival information was obtained through telephone follow-up.

### 2.7 Statistical analysis

Numerical data were presented as mean ± SD. Categorical data were presented as absolute frequency with relative frequency in parenthesis. The nonparametric Wilcoxon test and *t* test was used to compare paired data, χ2 test or Fisher’s exact test was used to analyze categorical data. Survival was compared by log-rank test and plotted as Kaplan-Meier diagrams. All *p* values were reported as two-tailed, with a significance level of 0.05. SPSS Statistics 22.0 and GraphPad Prism 9.3 were used for statistical analysis and mapping.

## 3 Results

### 3.1 Tumor and patient characteristics

**Figure 1:**
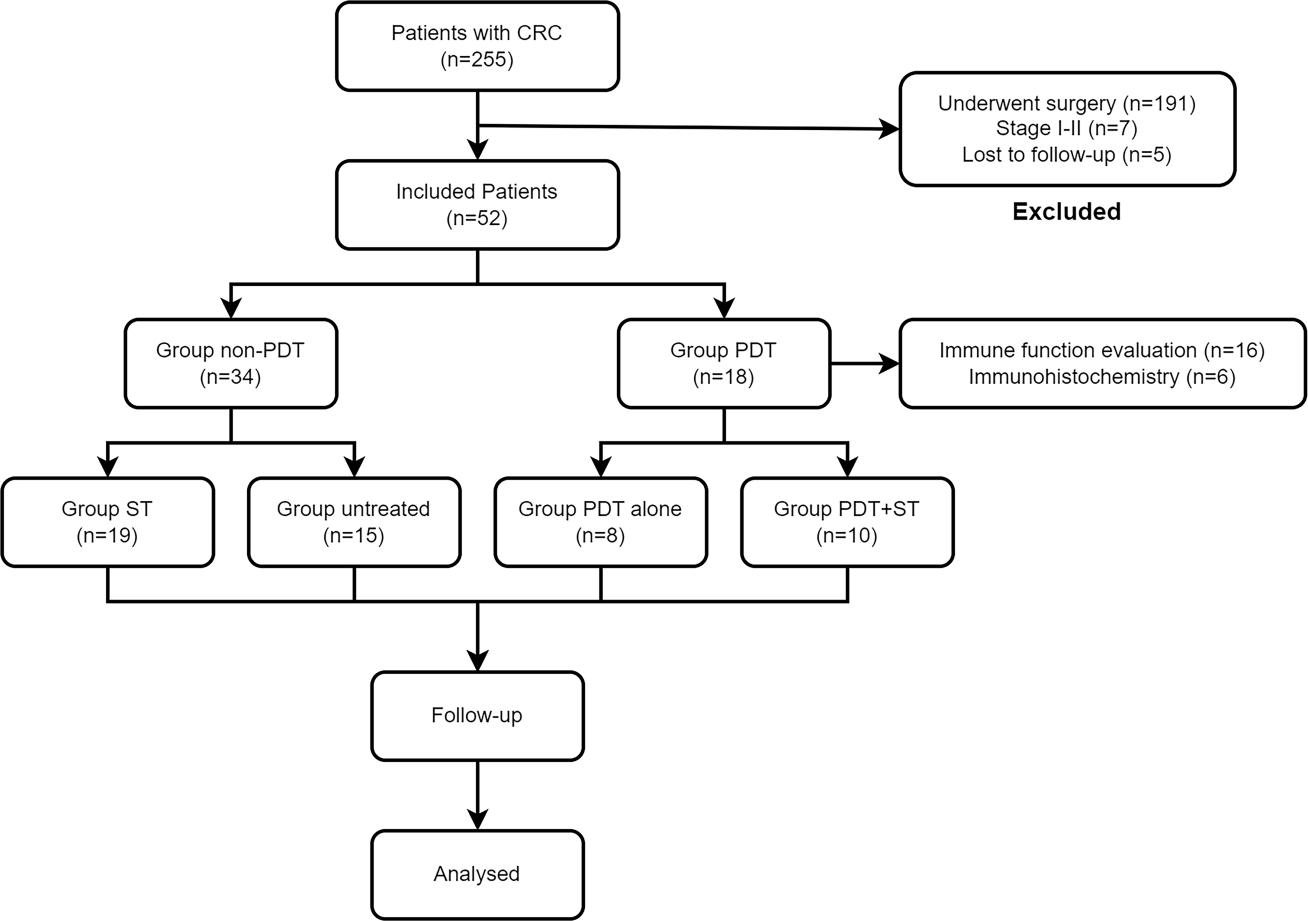
Flowchart of all patients in this study. CRC, colorectal cancer; PDT, photodynamic therapy; ST, systemic therapy.

A complete flow chart for all patients is presented in **Figure 1**. A total of 52 CRC patients were enrolled in this retrospective study from November 2020 to July 2021. **Table 1** lists the essential characteristics of the patients, including age, sex, location, degree of differentiation, clinical stage, KPS score, tumor length, and treatment plan. Eighteen patients received PDT and 34 did not. Rectal cancer was predominant in both groups, with 11 (61.1%) and 22 (64.7%) patients respectively. Regarding pathological type and degree of differentiation, moderately differentiated adenocarcinoma accounts for the highest proportion, followed by well-differentiated adenocarcinoma, and poorly differentiated adenocarcinoma is the lowest. Many patients had distant metastases at diagnosis, and the proportion was more than 50% in both groups (55.6% vs. 70.6%). Patients were differentiated by a KPS score of 70, divided into one group greater than 70 and another group less than 70, and the composition ratio of the KPS score was similar between the PDT group and the non-PDT group. Most patients had lesions longer than 4cm, meaning that patients in the PDT group often required segmental irradiation. Ten patients (55.6%) in the PDT group received systemic therapy, while 15 patients in the non-PDT group received no treatment after diagnosis. There were no significant differences between the two groups concerning all baseline parameters.

**Table 1:** Clinical characteristics of 52 CRC patients.

Clinical data	PDT group	Non-PDT group	*p*-value*
Number of patients (n)	18	34	
Age, years (mean ± SD)	58.0 ± 15.0	61.1 ± 11.5	0.425
Sex, n (%)			0.250
Male	12 (66.7)	17 (50.0)	
Female	6 (33.3)	17 (50.0)	
Location, n (%)			0.798
Colon	7 (38.9)	12 (35.3)	
Rectum	11 (61.1)	22 (64.7)	
Histologic differentiation, n (%)			0.843
W/D	6 (33.3)	10 (29.4)	
M/D	9 (50.0)	16 (47.1)	
P/D	3 (16.7)	8 (23.5)	
Clinical stage, n (%)			0.278
III	8 (44.4)	10 (29.4)	
IV	10 (55.6)	24 (70.6)	
KPS score, n (%)			0.488
≥70	7 (38.9)	10 (29.4)	
<70	11 (61.1)	24 (70.6)	
Length (cm), n (%)			0.369
≤4	4 (22.2)	6 (17.6)	
4<, ≤8	11 (61.1)	16 (47.1)	
>8	3 (16.7)	12 (35.3)	
Treatment regimen, n (%)			0.432
PDT alone	8 (44.4)	0	
PDT+ST	10 (55.6)	0	
ST	0	19 (55.9)	
Untreated	0	15 (44.1)	

*CRC, colorectal cancer; PDT, photodynamic therapy; W/D, well-differentiated; M/D, moderately differentiated; P/D, poorly differentiated; ST, systemic therapy.

### 3.2 Survival analysis

**Figure 2:**
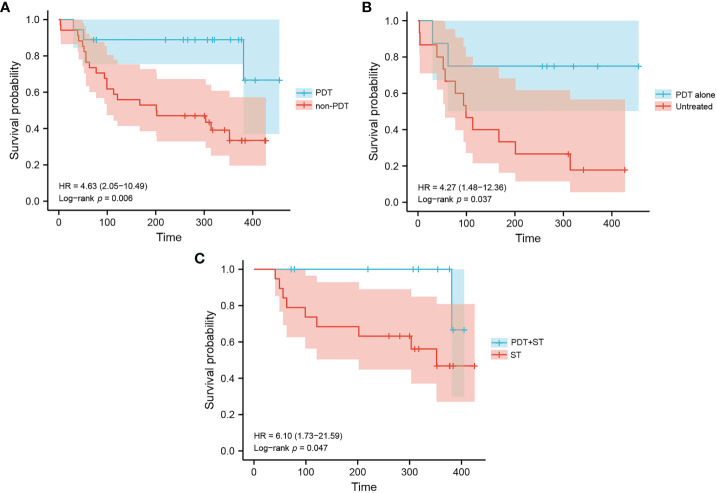
Kaplan-Meier estimation of patient survival in each group. **(A)** Overall survival of patients in the PDT group and non-PDT group. **(B)** Overall survival of patients in the PDT alone group and untreated group. **(C)** Overall survival of patients in the PDT+ST group and ST group.

Kaplan-Meier analysis of overall survivals (OS) in the PDT and non-PDT groups are shown in **Figures 2A–C**. Compared with non-PDT (median OS: 202 days), the PDT group (median OS: not reached) showed a longer OS (log-rank p=0.006) (**Figure 2A**). Subgroup analysis showed that PDT alone (median OS: not reached) had significantly longer OS compared to untreated patients (median OS: 99 days, log-rank p=0.037) (**Figure 2B**). Patients in the PDT+ST group (median OS: not reached) also had superior OS to those in the ST group (median OS: 352 days, log-rank p=0.047) (**Figure 2C**).

### 3.3 Short-term response

**Figure 3:**
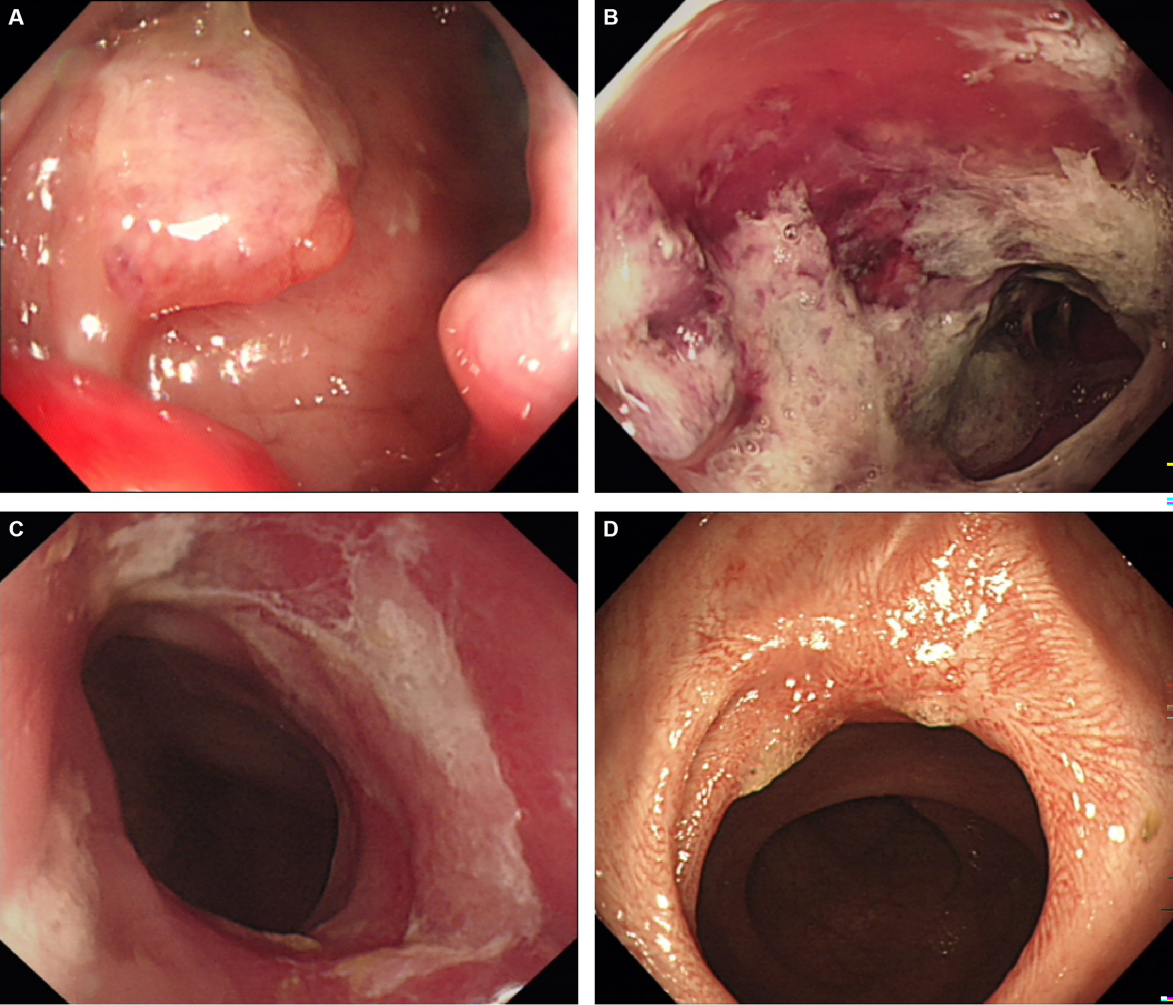
A representative case of achieving complete response after PDT. **(A)** The cauliflower-like protrusion was seen on the lateral wall of the rectum, and the clinical stage is stage IIIB; **(B)** 48h after PDT, a large amount of gray necrotic tissue was formed at the lesion site, with hyperemia and edema at the edge; **(C)** One month after PDT, the lesion’s surface was covered with a small amount of residual necrotic tissue, and the tumor almost disappeared; **(D)** Three months after PDT, the tumor tissue disappeared and a scar formed. Biopsy confirmed that the tumor cells were negative, so complete remission was achieved.

The short-term efficacy and possible influencing factors of PDT are shown in **Tables 2** **,** **3**. Of the 18 patients treated with PDT, 1 patient achieved complete response (CR), 7 patients achieved partial response (PR), 8 patients achieved stable disease (SD), and 2 patients died due to infection and other tumor-related adverse events. The objective response rate was 44.4%, and the disease control rate was 88.9% (**Table 3**). We further explored the possible causes affecting the short-term efficacy of PDT, and the degree of differentiation (*p*=0.020) and the degree of endoscopic necrosis (*p*=0.039) were two crucial factors (**Table 3**). Moderately differentiated had the best therapeutic effect, followed by well and poorly differentiated. The degree of necrosis under colonoscopy was positively correlated with short-term efficacy and positively correlated with short-term efficacy. More severe tumor necrosis means better short-term outcomes for patients. **Figure 3** shows a typical case of a patient who achieved complete remission of the local lesion shortly after diagnosing CRC with PDT.

**Table 2:** Clinical data for the eighteen CRC patients undergoing PDT.

Case	Stent	Clinical stage	Treatment regimen	KPS (Before)	KPS (After 2 mo)	Degree of necrosis (After 48h)	Short-term efficacy (After 2 mo)	Survival (Days)	Outcome
1	Yes	IIIC	PDT	70	80	Severe	SD	455	Alive
2	Yes	IVB	PDT+ST	70	90	Severe	PR	381	Death
3	No	IIIB	PDT+ST	80	90	Severe	CR	384	Alive
4	Yes	IIIC	PDT+ST	20	70	Moderate	SD	405	Alive
5	Yes	IVA	PDT	50	0	Moderate	PD	30	Death
6	Yes	IIIC	PDT+ST	60	80	Severe	SD	377	Alive
7	No	IIIC	PDT	60	70	Severe	PR	371	Alive
8	No	IVB	PDT+ST	60	80	Severe	PR	354	Alive
9	No	IVA	PDT	70	80	Severe	PR	321	Alive
10	Yes	IVC	PDT+ST	70	80	Moderate	PR	317	Alive
11	Yes	IIIC	PDT	30	0	Mild	PD	62	Death
12	No	IIIC	PDT+ST	60	70	Severe	SD	307	Alive
13	No	IVB	PDT	70	80	Moderate	SD	281	Alive
14	Yes	IVA	PDT	30	50	Moderate	SD	266	Alive
15	Yes	IVB	PDT	50	70	Severe	SD	257	Alive
16	Yes	IVC	PDT+ST	50	70	Moderate	PR	220	Alive
17	Yes	IVA	PDT+ST	70	80	Severe	SD	72	Alive
18	Yes	IIIC	PDT+ST	60	80	Severe	PR	78	Alive

ST, systemic therapy; CR, complete response; PR, partial response; SD, stable disease; PD, progressive disease.

**Table 3:** Short-term efficacy and the efficacy comparison of different types.

		Short-term efficacy				Objective response rate (%)	Disease control rate (%)	Invalid rate (%)			
Types	n	CR	PR	SD	PD	(CR+PR)/n	(CR+PR+SD)/n	PD/n	CR+PR+SD	PD	*p*-value*
**All patients**	18	1	7	8	2	44.4	88.9	11.1	16	2	
**Location**											0.641
Colon	7	0	3	3	1	42.9	85.7	14.3	6	1	
Rectum	11	1	4	5	1	45.5	90.9	9.1	10	1	
**Differentiation**											0.020
W/D	6	0	1	5	0	16.7	100	0	6	0	
M/D	9	1	5	3	0	66.7	100	0	9	0	
P/D	3	0	1	0	2	33.3	33.3	66.7	1	2	
**Clinical stage**											1.000
III	8	1	2	4	1	37.5	87.5	12.5	7	1	
IV	10	0	5	4	1	50	90	10	9	1	
**KPS score**											0.491
≥70	7	1	3	3	0	57.1	100	0	7	0	
<70	11	0	4	5	2	36.4	81.8	18.2	9	2	
**Length (cm)**											1.000
≤4	4	1	2	1	0	75	100	0	4	0	
<4 ≤ 8	11	0	3	6	2	27.3	81.8	18.2	9	2	
>8	3	0	2	1	0	66.7	100	0	3	0	
**Stent**											0.529
Yes	12	0	4	6	2	33.3	83.3	16.7	10	2	
No	6	1	3	2	0	66.7	100	0	6	0	
**Treatment line**											0.183
PDT	8	0	2	4	2	25	75	25	6	2	
PDT+ST	10	1	5	4	0	60	100	0	10	0	
**Necrosis**							0				
Mild	1	0	0	0	1	0	0	100	0	1	0.039
Moderate	6	0	2	3	1	33.3	83.3	16.7	5	1	
Severe	11	1	5	5	0	54.5	100	0	11	0	

*W/D, well-differentiated; M/D, moderately differentiated; P/D, poorly differentiated; ST, systemic therapy; CR, complete response; PR, partial response; SD, stable disease; PD, progressive disease.

### 3.4 Immune evaluation

**Figure 4:**
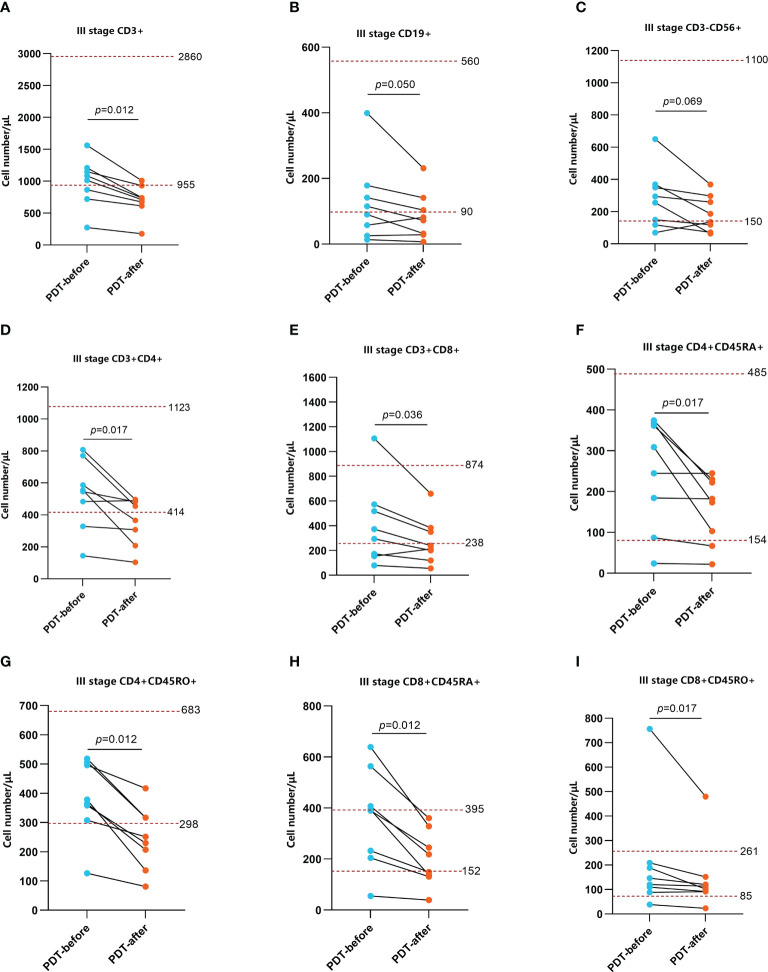
Changes in peripheral blood immune function before and after 48h of PDT in stage III CRC patients (n=8). The number of CD3^+^ T cells, CD3^+^CD4^+^ T cells, CD3^+^CD8^+^ T cells, CD4^+^CD45RA^+^ T cells, CD4^+^CD45RO^+^ T cells, CD8^+^CD45RA^+^ T cells, CD8^+^CD45RO^+^ T cells were basically at a normal level before PDT but significantly decreased at 48h after PDT **(A, D, E–I)**. CD19^+^ B cells and CD3^-^CD56^+^ NK cells were at lower than normal levels. Although there was no statistical difference in CD cells, the overall trend was still downward **(B, C)**.

**Figure 5:**
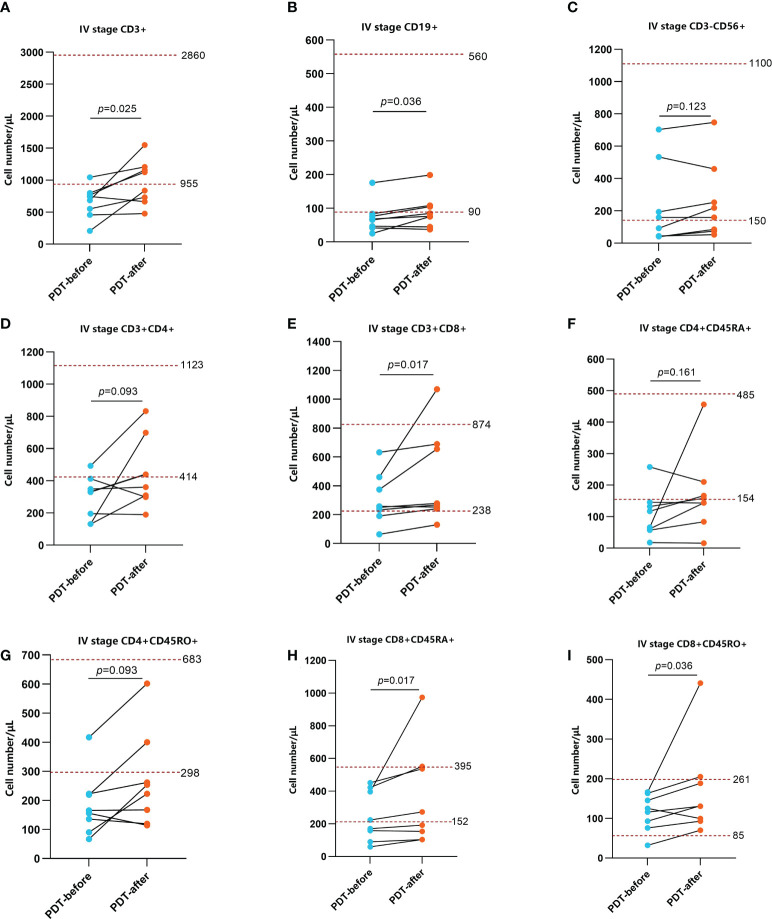
Changes in peripheral blood immune function in stage IV patients before and after PDT 48h (n=8). The number of CD3^+^ T cells, CD19^+^ B cells, CD3^+^CD8^+^ T cells, CD8^+^CD45RA^+^ T cells, CD8^+^CD45RO^+^ T cells were relatively lower before PDT and increased significantly 48h after PDT **(A, B, E, H, I)**. The changes of CD3^-^CD56^+^ NK cells, CD3^+^CD4^+^ T cells, CD4^+^CD45RA^+^ T cells CD4^+^CD45RO^+^ T cells were not obvious **(C, D, F, G)**.

To explore the effect of PDT on the immune microenvironment of CRC patients, we examined the changes of immune cells in peripheral blood and tumor tissues of patients receiving PDT. Detailed data from peripheral blood tests are shown in **Supplementary Tables S1**, **2**. The results before and after PDT are shown in **Figures 4**, **5**. Before and after PDT, the systemic immune cell numbers of stage III patients and stage IV patients showed an opposite trend. The former showed a majority decrease after PDT, while the latter showed an overall increase. Specifically, in 8 patients with stage III CRC, Total T cells, CD4^+^ T cells, CD8^+^ T cells, CD4^+^CD45RA^+^ T cells, CD4^+^CD45RO^+^ T cells, CD8^+^CD45RA^+^ T cells Cells, and CD4^+^CD45RO^+^ T cells were significantly decreased compared with those before PDT. B cells and NK cells were also decreased in most cases, although there was no statistical difference (**Figure 4**). In patients with stage IV, however, most of the immune cells increased after PDT. Among them are T cells, B cells, CD8^+^T cells, CD8^+^CD45RA^+^T cells, and CD4^+^CD45RO^+^T cells (**Figure 5**).

**Figure 6:**
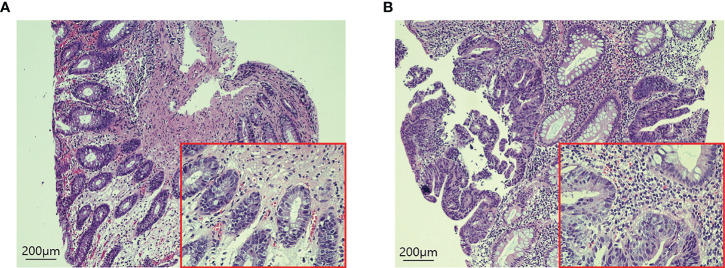
H-E staining of tumor tissues in CRC patients before and 48 hours after PDT. **(A)** Before PDT, a small number of immune cells were infiltrated around the tumor tissue. **(B)** 48h after PDT, the number of immune cells in tumor tissue increased significantly.

**Figure 7:**
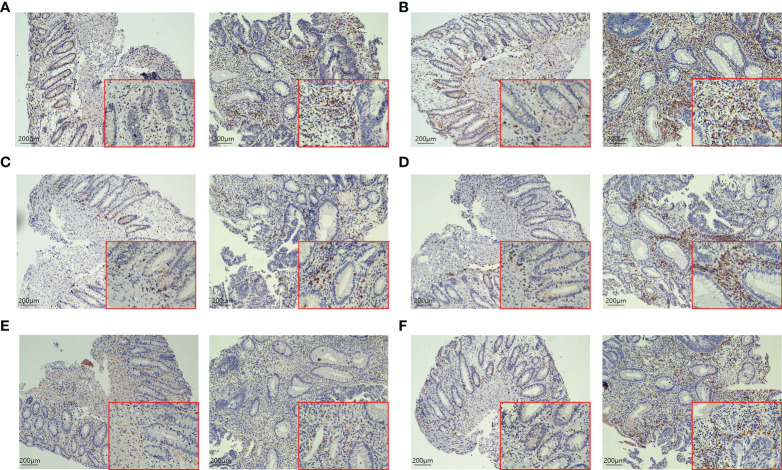
IHC staining of tumor tissues in CRC patients before and after 48h of PDT (n=6). Images are shown at 20× magnification; bars indicate 200 µm; the left is before PDT, and the right is 48h after PDT. **(A)** CD3^+^ T cells in tumor tissue were increased significantly after PDT. **(B)** The number of CD4^+^ T cells in tumor tissue was increased after PDT. **(C)** Changes in the number of CD8^+^ T cells in tumor tissues. CD8^+^ T cells were increased after PDT. **(D)** The number of CD20^+^ B cells goes up after PDT. **(E)** There was no significant change in CD56^+^ T cells. **(F)** The number of macrophages increased significantly after PDT.

H-E staining showed that many inflammatory cells and immune cells infiltrated the tumor tissue 48h after PDT (**Figure 6**). Immunohistochemical results showed that T cells (*p*=0.0053), B cells (*p*=0.0216), CD4^+^ T cells (*p*=0.0341), CD8^+^ T cells (*p*=0.0132), and macrophages (*p*=0.0172) increased significantly after PDT, while the number of NK cells (*p*=0.3276) did not change significantly (**Figure 7**; **Supplementary Figure S1**).

### 3.5 Adverse reactions

The distribution of adverse reactions after PDT is shown in **Table 4**. Transient leukocytosis occurred in 2 patients, and fever occurred in 3 patients on the night of treatment and returned to normal after physical cooling. One patient developed abdominal pain shortly after treatment and improved after rest. After discharge, 2 patients did not comply with the strict requirements of avoiding light and developed pigmentation of the skin on the face, forearm, and back of the hand. None of the patients had severe complications such as hematochezia and perforation.

**Table 4:** Toxicities related to PDT.

Events	Number	Rate (%)
WBC increase	2	11.1
Fever	3	16.7
Abdominal pain	1	5.6
Skin phototoxicity	2	11.1
Hematochezia	0	0
Perforation	0	0

WBC, white blood cells.

## 4 Discussion

Despite the increasing availability of an arsenal of anti-tumor weapons, non-specificity, drug resistance, low response rates, and toxic severe side effects are still the main challenges faced by current tumor treatment strategies (11). As an alternative intervention to destroy cancer cells, PDT can compensate for some deficiencies associated with conventional tumor therapy (12). In addition to the precise clinical efficacy, PDT also has the advantages of less pain, high acceptance, and repeatable operation. For some early or occult tumors, PDT may achieve the effect of a radical cure. For patients with advanced cancers or patients who cannot undergo surgery due to physical limitations, PDT is a palliative treatment that can effectively relieve pain, improve quality of life and prolong life (8). Clinical studies on PDT therapy in CRC are rarely seen compared with other gastrointestinal tumors. In this study, 52 CRC patients with advanced stages (III-A to IV-C) were included, all unable or unwilling to undergo surgical resection, including 18 patients receiving PDT and 34 patients not receiving PDT. More than 50% of patients have distant metastasis, indicating a poor prognosis (4), especially without any treatment.

We made a detailed comparison between the PDT group and the non-PDT group. The current short-term follow-up results show that the overall prognosis of the PDT group is better than that of the non-PDT group (*p*=0.006). Among them, the median survival time of the non-PDT group was only 6.7 months, while the median survival time of patients receiving PDT at the same time had not yet reached, which was encouraging. Comparing CRC patients who received PDT alone with those who did not receive any treatment, the results were also gratifying in this case (*p*=0.037). Cause PDT can directly kill tumor cells and eliminate tumors by destroying the microcirculation of tumor tissues and enhancing the body’s anti-tumor immunity, thus prolonging the survival time of patients (13–15). Some patients with CRC received PDT combined with other therapies, such as chemotherapy, immunotherapy, or targeted therapy. Compared with patients who only received other treatments, the survival rate of the PDT combined with other therapies was significantly better (*p*=0.047), which may be due to the synergistic reaction between PDT and other therapies (16, 17). Unfortunately, one patient died of severe infection and multiple organ failures due to multiple liver and lung metastases. The above results showed that PDT alone or combined with other therapies showed significant advantages. However, the number of patients receiving PDT in each subgroup was small, and the survival data were only exploratory. However, the pilot series contains a minimal number of patients, and the survival data are only preliminary experimental.

To assess the direct efficacy of PDT, we evaluated the short-term efficacy of patients with CRC who had completed PDT by endoscopic and radiographic examinations after two months, Taking RECIST version 1.1 as the judge. Not surprisingly, the therapeutic effect of PDT was rapid and pronounced, especially in the remission of local lesions. In fact, PDT continues to play a therapeutic role. Many patients could still see pieces of necrotic tissue falling off the original tumor site when they were reviewed by colonoscopy one month later. Objective response was achieved in nearly half of the 18 CRC patients (8/18), and the disease control rate was 88.9% (16/18). By analyzing the factors that might affect the short-term efficacy of PDT, we found two valuable indicators: endoscopic necrosis degree and tumor differentiation degree. Obviously, the more severe the necrosis, the better the treatment effect of PDT. This process includes apoptosis, regulated necrosis (such as necroptosis and lysosome-dependent cell death), and other cell death mechanisms (18, 19). Due to the limited penetration depth of the laser in tissues (5-7mm), large and deeply infiltrated tumors could not be eliminated at a time. Therefore, the complete remission rate of this group of cases was only 5.6% (1/18). The short-term efficacy of PDT was also correlated with differentiation degree. Moderately differentiated colorectal tumors performed best, mainly because the tumor tissues were enriched in photosensitizers and had sufficient blood supply, and the objective response rate reached 66.7% (6/9); the reason may be that the characteristics of well-differentiation tumors are close to normal tissues, and the enrichment of photosensitizer in tumor tissues is almost the same as that in normal tissues, so the efficacy of PDT is not ideal (20, 21). Patients with poorly differentiation have a higher degree of malignancy and faster disease progression, resulting in a relative lack of blood supply as well as insufficient free radical production, thus the effect is not good.

The changes in the immune function of patients before and after PDT are a focus of our attention. Interestingly, the systemic immune function of almost all patients with stage III CRC decreased compared to before PDT. In contrast, the immune indicators of stage IVpatients generally showed an upward trend. Further observation illustrated that the number of immune cells in patients with stage III remained normal or slightly low before PDT, while the initial immune function of patients with stage IV was decreased. We speculate that this change is that a mass of immune cells quickly gathered at the tumor site, decreasing blood content. However, since the total amount of various types of immune cells was not low before the intervention, the overall level still fluctuated around the lower limit of the normal range, which may be a time difference problem. CRC patients with stage III are generally in good condition, basically normal organ function, well nutritional status with near-normal peripheral blood immune cells. The peripheral immune cells rapidly reach the local tumor lesions after antigen is released from necrotic tumor cells, decreasing peripheral immune cell numbers after PDT. However, due to distant metastasis, organ dysfunction, malnutrition, and cachexia, the immune cells of stage IV patients have been partially depleted, and the initial immune function in the body is relatively deficient. PDT intervention, on the one hand, can stimulate immune organs to generate more immune cells and improve the body’s overall immune level, causing an increase in peripheral immune cell numbers after PDT; on the other hand, it can promote the local recruitment of systemic immune cells to tumor tissues and play an anti-tumor immune role. To test this idea, we detected the changes of immune cells in tumor tissues before and after PDT by IHC. The results showed that a large number of inflammatory cells and immune cells were infiltrated in the tumor tissues of almost all CRC patients. In addition, NK cells, T cells, B cells, CD4^+^ T cells, CD8^+^ T cells, and macrophages were markedly increased, which provided an answer for the decline of immune cells in the peripheral blood of stage III patients.

These processes indicate that PDT is likely to trigger immunogenic cell death (ICD) through chemical damage, recruit DCs and antigen-specific cytotoxic T lymphocytes (CTLs) to the tumor microenvironment (TME), reverse the “cold tumor” to “hot tumor”, and activate the anti-tumor immune response. In addition to killing *in situ* tumors, it can effectively inhibit distal and metastatic tumors by improving the survival rate of patients with malignant tumors (22, 23). Once the signal of photo-oxidative damage caused by PDT is sensed, neutrophils are the first to reach the tumor site and subsequently direct a large number of monocytes/macrophages to rapidly invade the PDT-irradiated tumor margin to remove injured and dead cells (24, 25). In contrast, studies have shown that the therapeutic effect of PDT is significantly reduced if the above inflammatory cells are absent or their activity is inhibited (26). Acute inflammatory response increases the presentation of tumor antigens, paving the way for subsequent adaptive immunity (8). Studies have shown that PDT intervention enhances the activation of tumor-specific CD8^+^ T cells (26, 27). In the absence of CD8^+^ T cell activation and/or tumor invasion, the efficacy of PDT is significantly reduced (28), suggesting that CD8^+^ T cells play a central role in PDT-induced anti-tumor immunity. In most cases, the CD8^+^ effect and memory T cell generation depend on the presence and activation of CD4^+^ T cells. Earlier studies have shown that immune deficiency of helper T cells leads to a significant reduction in PDT-mediated tumor healing (29). However, some studies reveal that CD4^+^ T cells play a limited role in PDT therapy (28). In this study, CD4^+^ T cells increased significantly after treatment, which may help CD8^+^ T cells function.

In addition to immune cells, PDT may also affect stromal components of the TME. Fibroblasts constitute one of the most important cells in the stroma. They play an active role in the initiation and progression of various tumors, and shape the TME by directly inhibiting anti-tumor immune response or recruiting immunosuppressive cells (30). On the one hand, some studies have shown that PDT may reverse the fibroblast-myofibroblast differentiation and reshape the extracellular matrix (ECM) (31). However, others believe that PDT has no apparent cytotoxicity to fibroblasts (32), further research must be revealed. On the other hand, the effect of PDT is less affected by the therapeutic resistance induced by carcinoma-associated fibroblasts (CAFs) (33).

Of course, it is difficult to eliminate all tumor cells by PDT alone, especially in the case of distant metastasis. The combination of PDT with other therapies has considerable appeal in terms of enhancing efficacy against tumors because it exploits the advantages and offsets the disadvantages of each treatment method to produce additional or even synergistic therapeutic effects (34, 35). Moreover, using lower doses in combination therapy may cause fewer side effects and better results than single therapy (35). Chemotherapy relieves the restriction of light penetration in PDT and enhances the sensitivity of cancer cells to reactive oxygen species. The broad-spectrum activity and non-drug resistance of PDT can also combat the troublesome problem of drug resistance in chemotherapy (36, 37). Chemotherapy and PDT are combined in mutual assistance has significantly improved the clinical efficacy compared with single PDT or chemotherapy (38). The combination of PDT and immunotherapy can effectively eradicate target tumors and possible residual cancer cells and metastases and trigger immune memory to prevent tumor recurrence and provide a possible cure (34). Some targeted drugs, such as anti-angiogenesis or anti-vascular drugs (39) and anti-epidermal growth factor receptor drugs (40), have also been proven to improve the efficacy of PDT further and significantly improve tumor growth control after PDT. In our study, one patient with low rectal cancer received chemotherapy combined with targeted and immunotherapy after one week of PDT. After three months of follow-up, a complete pathological complete response (pCR) was achieved.

The adverse reactions of the enrolled patients during PDT were similar to those reported in the literature (41), mainly transient leukocyte elevation and mild fever, which may be related to inflammatory reactions caused by treatment. Occasionally, patients may have mild abdominal pain, which can be relieved after rest. Other common adverse reactions include photoallergic reactions such as redness and pigmentation at the exposure site, associated with irregular light avoidance after PDT. Once it occurs, it can be improved by giving antiallergic drugs and topical corticosteroid scrubs.

Some limitations of this study need to be mentioned. First, this study is a single-center retrospective study, selection bias is inevitable, and other investigators’ findings are subject to external validation. Second, the short follow-up time and the absence of some outcome indicators may lead to the overestimation of the therapeutic effect of PDT. Third and most important, the relatively small number of patients limits our ability to perform statistical analyses, especially when comparing different subgroups. Further multicenter prospective randomized trials are needed to compare the effects of PDT on the CRC immune microenvironment and in combination with other measures.

## 5 Conclusions

In this study, PDT effectively relieved the obstructive symptoms of CRC patients, inhibited tumor growth, and prolonged the survival time of patients with reasonable safety. Subsequently, we further evaluated CRC patients’ systemic and tumor local immune microenvironment changes before and after PDT. PDT recruits various immune cells to surround tumors, transforms “cold tumors” into “hot tumors”, activates the body’s anti-tumor immune effect, and then exerts tumor-killing effects at local and distant sites. PDT has synergistic effects when combined with chemotherapy, immunotherapy, or targeted therapy. With the development of more clinical studies and more efficient photosensitizers, PDT will occupy a space to treat more tumors.

## Data availability statement

The original contributions presented in the study are included in the article/**Supplementary Material**. Further inquiries can be directed to the corresponding author.

## Ethics statement

The studies involving human participants were reviewed and approved by the Committee of Medical Ethics Experts of the Second Hospital of Lanzhou University. Written informed consent for participation was not required for this study in accordance with the national legislation and the institutional requirements.

## Author contributions

Study conception and design: BG, BW, and HC. Acquisition of data: BG, CM, LG, JZ, PZ, YW. Analysis and interpretation of data: BG, BW, XL, ZF. Statistical analysis: BG, BW, YY. Drafting the article: BG, BW, XL, ZF. Critical revision of the article: HL, TZ, HC. All authors contributed to the article and approved the submitted version.

## Funding

This research was supported by Key Project of Science and Technology in Gansu Province (19ZD2WA001), Key Talents Program of Gansu Province (2019RCXM020), Cuiying Scientific and Technological Innovation Program of Lanzhou University Second Hospital (CY2017-ZD01), Science and Technology Project of Chengguan District of Lanzhou City (2020SHFZ0039), Outstanding Doctoral Program of Natural Science Foundation of Gansu Province (22JR5RA945), “Innovation Star” Project for Outstanding Graduate Students in Gansu Province (2022CXZX-162).

## Conflict of interest

The authors declare that the research was conducted in the absence of any commercial or financial relationships that could be construed as a potential conflict of interest.

## Publisher’s note

All claims expressed in this article are solely those of the authors and do not necessarily represent those of their affiliated organizations, or those of the publisher, the editors and the reviewers. Any product that may be evaluated in this article, or claim that may be made by its manufacturer, is not guaranteed or endorsed by the publisher.

## Supplementary material

Supplementary Figure 1 Immune cell infiltration in tumor tissues of CRC patients before and after 48h PDT (n=6). The results showed that T cells (*p*=0.0053), B cells (*p*=0.0216), CD4^+^ T cells (*p*=0.0341), CD8^+^ T cells (*p*=0.0132), and macrophages (*p*=0.0172) increased significantly after PDT, and NK cells did not change much.

## References

[1] Sung H Ferlay J Siegel RL Laversanne M Soerjomataram I Jemal A. Global cancer statistics 2020: Globocan estimates of incidence and mortality worldwide for 36 cancers in 185 countries. CA Cancer J Clin (2021) 71(3):209–49. doi: 10.3322/caac.21660 33538338

[2] Zhou J Zheng R Zhang S Zeng H Wang S Chen R. Colorectal cancer burden and trends: Comparison between China and major burden countries in the world. Chin J Cancer Res (2021) 33(1):1–10. doi: 10.21147/j.issn.1000-9604.2021.01.01 33707923 PMC7941684

[3] Dekker E Tanis PJ Vleugels JLA Kasi PM Wallace MB. Colorectal cancer. Lancet (2019) 394(10207):1467–80. doi: 10.1016/s0140-6736(19)32319-0 31631858

[4] Siegel RL Miller KD Goding Sauer A Fedewa SA Butterly LF Anderson JC. Colorectal cancer statistics, 2020. CA Cancer J Clin (2020) 70(3):145–64. doi: 10.3322/caac.21601 32133645

[5] Battersby NJ Bouliotis G Emmertsen KJ Juul T Glynne-Jones R Branagan G. Development and external validation of a nomogram and online tool to predict bowel dysfunction following restorative rectal cancer resection: The polars score. Gut (2018) 67(4):688–96. doi: 10.1136/gutjnl-2016-312695 28115491

[6] Mishra J Drummond J Quazi SH Karanki SS Shaw JJ Chen B. Prospective of colon cancer treatments and scope for combinatorial approach to enhanced cancer cell apoptosis. Crit Rev Oncol Hematol (2013) 86(3):232–50. doi: 10.1016/j.critrevonc.2012.09.014 PMC3561496 23098684

[7] Kaleta-Richter M Kawczyk-Krupka A Aebisher D Bartusik-Aebisher D Czuba Z Cieślar G. The capability and potential of new forms of personalized colon cancer treatment: Immunotherapy and photodynamic therapy. Photodiagnosis Photodyn Ther (2019) 25:253–8. doi: 10.1016/j.pdpdt.2019.01.004 30611864

[8] Agostinis P Berg K Cengel KA Foster TH Girotti AW Gollnick SO. Photodynamic therapy of cancer: An update. CA Cancer J Clin (2011) 61(4):250–81. doi: 10.3322/caac.20114 PMC3209659 21617154

[9] Gollnick SO Vaughan L Henderson BW. Generation of effective antitumor vaccines using photodynamic therapy. Cancer Res (2002) 62(6):1604–8. 11912128

[10] Kimura M Miyajima K Kojika M Kono T Kato H. Photodynamic therapy (Pdt) with chemotherapy for advanced lung cancer with airway stenosis. Int J Mol Sci (2015) 16(10):25466–75. doi: 10.3390/ijms161025466 PMC4632810 26512656

[11] Simelane NWN Kruger CA Abrahamse H. Targeted nanoparticle photodynamic diagnosis and therapy of colorectal cancer. Int J Mol Sci (2021) 22(18):9779. doi: 10.3390/ijms22189779 34575942 PMC8466279

[12] Winifred Nompumelelo Simelane N Abrahamse H. Nanoparticle-mediated delivery systems in photodynamic therapy of colorectal cancer. Int J Mol Sci (2021) 22(22):12405. doi: 10.3390/ijms222212405 34830287 PMC8622021

[13] Kwiatkowski S Knap B Przystupski D Saczko J Kędzierska E Knap-Czop K. Photodynamic therapy - mechanisms, photosensitizers and combinations. BioMed Pharmacother (2018) 106:1098–107. doi: 10.1016/j.biopha.2018.07.049 30119176

[14] Spring BQ Rizvi I Xu N Hasan T. The role of photodynamic therapy in overcoming cancer drug resistance. Photochem Photobiol Sci (2015) 14(8):1476–91. doi: 10.1039/c4pp00495g PMC4520758 25856800

[15] van Straten D Mashayekhi V de Bruijn HS Oliveira S Robinson DJ. Oncologic photodynamic therapy: Basic principles, current clinical status and future directions. Cancers (Basel) (2017) 9(2):19. doi: 10.3390/cancers9020019 28218708 PMC5332942

[16] Hong MJ Cheon YK Lee EJ Lee TY Shim CS. Long-term outcome of photodynamic therapy with systemic chemotherapy compared to photodynamic therapy alone in patients with advanced hilar cholangiocarcinoma. Gut Liver (2014) 8(3):318–23. doi: 10.5009/gnl.2014.8.3.318 PMC4026651 24827630

[17] Nkune NW Kruger CA Abrahamse H. Possible enhancement of photodynamic therapy (Pdt) colorectal cancer treatment when combined with cannabidiol. Anticancer Agents Med Chem (2021) 21(2):137–48. doi: 10.2174/1871520620666200415102321 32294046

[18] Bacellar IO Tsubone TM Pavani C Baptista MS. Photodynamic efficiency: From molecular photochemistry to cell death. Int J Mol Sci (2015) 16(9):20523–59. doi: 10.3390/ijms160920523 PMC4613217 26334268

[19] Kessel D. Death pathways associated with photodynamic therapy. Med Laser Appl (2006) 21(4):219–24. doi: 10.1016/j.mla.2006.05.006 PMC2772082 19890442

[20] Allison RR Sibata CH. Oncologic photodynamic therapy photosensitizers: A clinical review. Photodiagnosis Photodyn Ther (2010) 7(2):61–75. doi: 10.1016/j.pdpdt.2010.02.001 20510301

[21] Juzeniene A Peng Q Moan J. Milestones in the development of photodynamic therapy and fluorescence diagnosis. Photochem Photobiol Sci (2007) 6(12):1234–45. doi: 10.1039/b705461k 18046478

[22] Fan Z Liu H Xue Y Lin J Fu Y Xia Z. Reversing cold tumors to hot: An immunoadjuvant-functionalized metal-organic framework for multimodal imaging-guided synergistic photo-immunotherapy. Bioact Mater (2021) 6(2):312–25. doi: 10.1016/j.bioactmat.2020.08.005 PMC7475520 32954050

[23] Li Z Zhu L Sun H Shen Y Hu D Wu W. Fluorine assembly nanocluster breaks the shackles of immunosuppression to turn the cold tumor hot. Proc Natl Acad Sci USA (2020) 117(52):32962–9. doi: 10.1073/pnas.2011297117 PMC7780000 33318219

[24] Dougherty TJ Gomer CJ Henderson BW Jori G Kessel D Korbelik M. Photodynamic therapy. J Natl Cancer Inst (1998) 90(12):889–905. doi: 10.1093/jnci/90.12.889 9637138 PMC4592754

[25] Krosl G Korbelik M Dougherty GJ. Induction of immune cell infiltration into murine sccvii tumour by photofrin-based photodynamic therapy. Br J Cancer (1995) 71(3):549–55. doi: 10.1038/bjc.1995.108 PMC2033617 7880738

[26] Kousis PC Henderson BW Maier PG Gollnick SO. Photodynamic therapy enhancement of antitumor immunity is regulated by neutrophils. Cancer Res (2007) 67(21):10501–10. doi: 10.1158/0008-5472.Can-07-1778 PMC2919236 17974994

[27] Mroz P Vatansever F Muchowicz A Hamblin MR. Photodynamic therapy of murine mastocytoma induces specific immune responses against the Cancer/Testis antigen P1a. Cancer Res (2013) 73(21):6462–70. doi: 10.1158/0008-5472.Can-11-2572 PMC3831658 24072749

[28] Kabingu E Vaughan L Owczarczak B Ramsey KD Gollnick SO. Cd8+ T cell-mediated control of distant tumours following local photodynamic therapy is independent of Cd4+ T cells and dependent on natural killer cells. Br J Cancer (2007) 96(12):1839–48. doi: 10.1038/sj.bjc.6603792 PMC2359961 17505510

[29] Korbelik M Cecic I. Contribution of myeloid and lymphoid host cells to the curative outcome of mouse sarcoma treatment by photodynamic therapy. Cancer Lett (1999) 137(1):91–8. doi: 10.1016/s0304-3835(98)00349-8 10376798

[30] Gok Yavuz B Gunaydin G Kosemehmetoglu K Karakoc D Ozgur F Guc D. The effects of cancer-associated fibroblasts obtained from atypical ductal hyperplasia on anti-tumor immune responses. Breast J (2018) 24(6):1099–101. doi: 10.1111/tbj.13139 30264475

[31] Zhang C Wang J Chou A Gong T Devine EE Jiang JJ. Photodynamic therapy induces antifibrotic alterations in primary human vocal fold fibroblasts. Laryngoscope (2018) 128(9):E323–e31. doi: 10.1002/lary.27219 29668038

[32] Gomes-Filho JE Sivieri-Araujo G Sipert CR da Silva Santos LM de Azevedo Queiroz ÍO Men Martins C. Evaluation of photodynamic therapy on fibroblast viability and cytokine production. Photodiagnosis Photodyn Ther (2016) 13:97–100. doi: 10.1016/j.pdpdt.2016.01.007 26796031

[33] Chen YC Lou X Zhang Z Ingram P Yoon E. High-throughput cancer cell sphere formation for characterizing the efficacy of photo dynamic therapy in 3d cell cultures. Sci Rep (2015) 5:12175. doi: 10.1038/srep12175 26153550 PMC4495468

[34] Li X Lovell JF Yoon J Chen X. Clinical development and potential of photothermal and photodynamic therapies for cancer. Nat Rev Clin Oncol (2020) 17(11):657–74. doi: 10.1038/s41571-020-0410-2 32699309

[35] Gunaydin G Gedik ME Ayan S. Photodynamic therapy for the treatment and diagnosis of cancer-a review of the current clinical status. Front Chem (2021) 9:686303. doi: 10.3389/fchem.2021.686303 34409014 PMC8365093

[36] He C Liu D Lin W. Self-assembled core-shell nanoparticles for combined chemotherapy and photodynamic therapy of resistant head and neck cancers. ACS Nano (2015) 9(1):991–1003. doi: 10.1021/nn506963h 25559017

[37] Wang Z Ma R Yan L Chen X Zhu G. Combined chemotherapy and photodynamic therapy using a nanohybrid based on layered double hydroxides to conquer cisplatin resistance. Chem Commun (Camb) (2015) 51(58):11587–90. doi: 10.1039/c5cc04376j 26096645

[38] Chen Y Zhang L Li F Sheng J Xu C Li D. Combination of chemotherapy and photodynamic therapy with oxygen self-supply in the form of mutual assistance for cancer therapy. Int J Nanomedicine (2021) 16:3679–94. doi: 10.2147/ijn.S298146 PMC8169060 34093012

[39] Ferrario A von Tiehl KF Rucker N Schwarz MA Gill PS Gomer CJ. Antiangiogenic treatment enhances photodynamic therapy responsiveness in a mouse mammary carcinoma. Cancer Res (2000) 60(15):4066–9. 10945611

[40] Cengel KA Hahn SM Glatstein E. C225 and pdt combination therapy for ovarian cancer: The play's the thing. J Natl Cancer Inst (2005) 97(20):1488–9. doi: 10.1093/jnci/dji360 16234556

[41] Zeng R Liu C Li L Cai X Chen R Li Z. Clinical efficacy of hiporfin photodynamic therapy for advanced obstructive esophageal cancer. Technol Cancer Res Treat (2020) 19:1533033820930335. doi: 10.1177/1533033820930335 32578508 PMC7315671

