# Supplementary figures and images for: Photodynamic therapy improves the clinical efficacy of advanced colorectal cancer and recruits immune cells into the tumor immune microenvironment
Source: Front Immunol. 2022 Nov 17;13:1050421. doi: 10.3389/fimmu.2022.1050421 (PMC9716470; doi:10.3389/fimmu.2022.1050421)

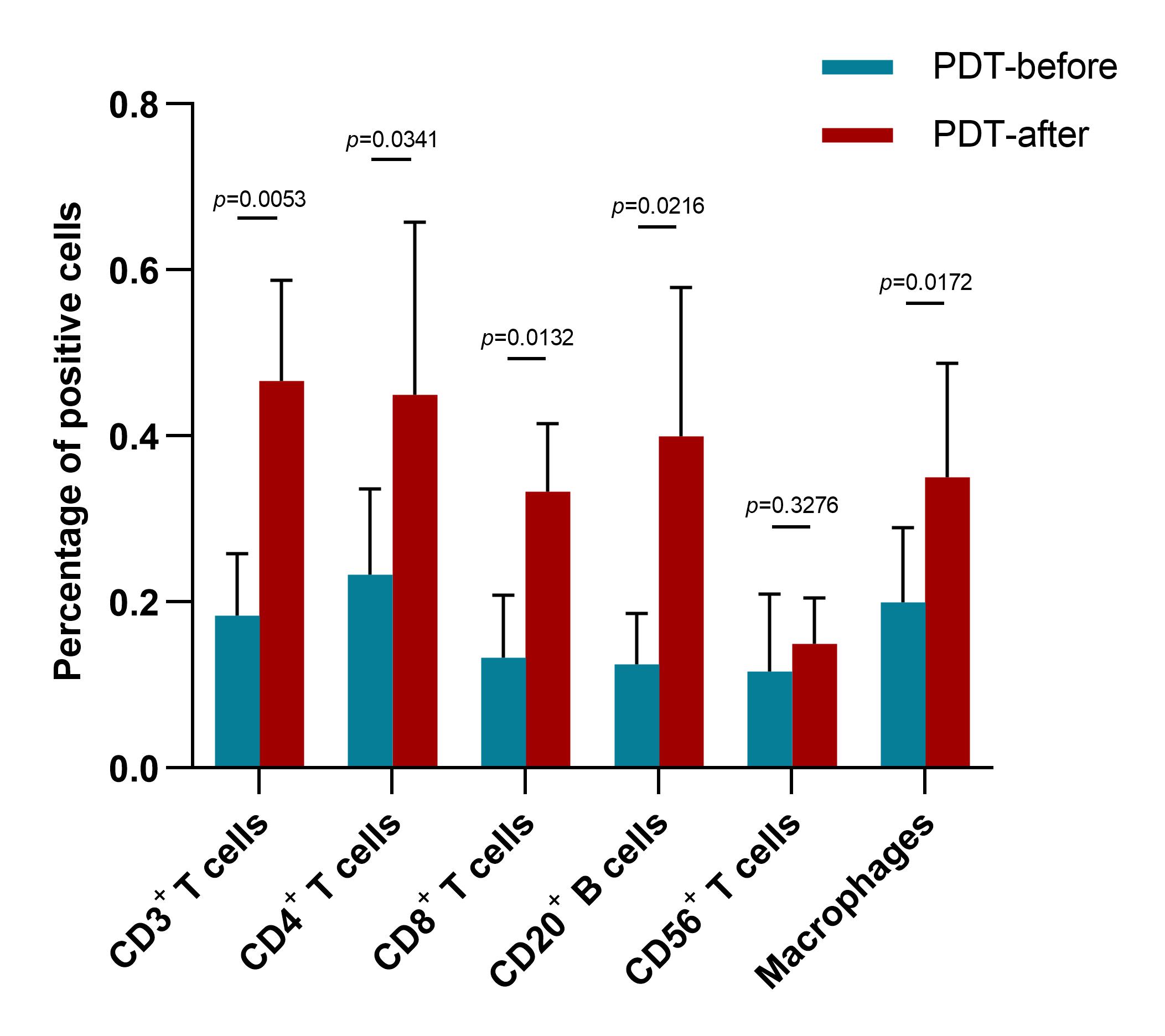

Supplement: Supplementary Figure 1 — Immune cell infiltration in tumor tissues of CRC patients before and after 48h PDT (n=6). The results showed that T cells (p=0.0053), B cells (p=0.0216), CD4+ T cells (p=0.0341), CD8+ T cells (p=0.0132), and macrophages (p=0.0172) increased significantly after PDT, and NK cells did not change much. [file Image_1.jpeg]
