# Supplementary material for: Photodynamic therapy improves the clinical efficacy of advanced colorectal cancer and recruits immune cells into the tumor immune microenvironment
Source: Front Immunol. 2022 Nov 17;13:1050421. doi: 10.3389/fimmu.2022.1050421 (PMC9716470; doi:10.3389/fimmu.2022.1050421)
Supplement: Supplementary file 2 [file Table_1.docx]

**Supplementary Table S1**

**Supplementary Table S1**. Results of peripheral blood immune cell detection in Ⅲ stage CRC patients.

| Case | CD3+ | | CD19+ | | CD3-CD56+ | | CD3+CD4+ | | CD3+CD8+ | | CD4+CD45RA+ | | CD4+CD45RO+ | | CD8+CD45RA+ | | CD8+CD45RO+ | |
| --- | --- | --- | --- | --- | --- | --- | --- | --- | --- | --- | --- | --- | --- | --- | --- | --- | --- | --- |
|  | PDT before | PDT after | PDT before | PDT after | PDT before | PDT after | PDT before | PDT after | PDT before | PDT after | PDT before | PDT after | PDT before | PDT after | PDT before | PDT after | PDT before | PDT after |
| 1 | 1014.6 | 711.55 | 399 | 231.42 | 294.5 | 259.35 | 587.1 | 365.75 | 372.4 | 239.4 | 184.3 | 182.21 | 507.3 | 316.54 | 389.5 | 244.72 | 209 | 151.62 |
| 2 | 1084.38 | 739.86 | 141.51 | 103.84 | 149.46 | 118 | 771.15 | 455.48 | 154.23 | 212.4 | 367.29 | 173.46 | 496.08 | 416.54 | 232.14 | 148.68 | 146.28 | 120.36 |
| 3 | 1561.37 | 1009.36 | 90.37 | 32.24 | 69.48 | 135.16 | 555.84 | 208.32 | 1105.89 | 659.68 | 308.8 | 102.92 | 378.28 | 136.4 | 407.23 | 218.24 | 756.56 | 479.88 |
| 4 | 720.02 | 613.20 | 115.37 | 72.45 | 368.35 | 186.90 | 546.27 | 483.00 | 172.36 | 119.70 | 361.4 | 229.95 | 364.18 | 206.85 | 204.33 | 131.25 | 109.81 | 91.35 |
| 5 | 1148.40 | 931.70 | 57.60 | 81.62 | 349.20 | 297.22 | 484.20 | 489.72 | 572.40 | 383.46 | 244.80 | 244.86 | 307.80 | 251.02 | 639.00 | 328.02 | 88.20 | 90.86 |
| 6 | 274.86 | 176.76 | 13.5 | 7.2 | 117.72 | 71.28 | 144.72 | 104.04 | 78.84 | 55.80 | 23.76 | 21.96 | 126.36 | 81 | 55.08 | 38.88 | 38.34 | 22.68 |
| 7 | 1209.6 | 743.04 | 178.56 | 141.48 | 255.36 | 63.72 | 808.32 | 495.72 | 293.76 | 200.88 | 374.40 | 222.48 | 518.4 | 316.44 | 393.6 | 137.16 | 188.16 | 101.52 |
| 8 | 866.8 | 673.34 | 25.61 | 28.82 | 650.1 | 368.11 | 328.99 | 307.85 | 518.11 | 349.77 | 86.68 | 66.81 | 358.54 | 229.25 | 563.42 | 360.25 | 120.17 | 113.97 |
