# Supplementary material for: Photodynamic therapy improves the clinical efficacy of advanced colorectal cancer and recruits immune cells into the tumor immune microenvironment
Source: Front Immunol. 2022 Nov 17;13:1050421. doi: 10.3389/fimmu.2022.1050421 (PMC9716470; doi:10.3389/fimmu.2022.1050421)
Supplement: Supplementary file 3 [file Table_2.docx]

**Supplementary Table S2**

**Supplementary Table S2**. Results of peripheral blood immune cell detection in Ⅳ stage CRC patients.

| Case | CD3+ | | CD19+ | | CD3-CD56+ | | CD3+CD4+ | | CD3+CD8+ | | CD4+CD45RA+ | | CD4+CD45RO+ | | CD8+CD45RA+ | | CD8+CD45RO+ | |
| --- | --- | --- | --- | --- | --- | --- | --- | --- | --- | --- | --- | --- | --- | --- | --- | --- | --- | --- |
|  | PDT before | PDT after | PDT before | PDT after | PDT before | PDT after | PDT before | PDT after | PDT before | PDT after | PDT before | PDT after | PDT before | PDT after | PDT before | PDT after | PDT before | PDT after |
| 1 | 1044.37 | 1205.82 | 76.02 | 104.94 | 533.95 | 459.36 | 329.42 | 439.56 | 631.69 | 689.04 | 132.13 | 158.4 | 222.63 | 261.36 | 450.69 | 536.58 | 115.84 | 130.68 |
| 2 | 205.15 | 835.17 | 25.3 | 75.03 | 159.5 | 159.90 | 131.45 | 698.64 | 63.8 | 130.38 | 66.00 | 456.33 | 66.55 | 253.38 | 58.85 | 103.32 | 31.9 | 70.11 |
| 3 | 455.7 | 476.64 | 41.54 | 37.44 | 44.64 | 52.56 | 195.30 | 189.36 | 250.48 | 278.64 | 17.98 | 15.84 | 155.62 | 113.76 | 89.90 | 103.68 | 125.24 | 99.36 |
| 4 | 684.32 | 1549.6 | 83.66 | 108.16 | 92.12 | 218.4 | 130.66 | 309.92 | 460.6 | 1069.12 | 61.1 | 143.53 | 90.24 | 222.56 | 395.74 | 973.44 | 166.38 | 440.96 |
| 5 | 798 | 1123.20 | 46.74 | 44.55 | 41.04 | 74.25 | 348.84 | 360.45 | 373.92 | 654.75 | 145.92 | 143.1 | 165.3 | 167.4 | 421.8 | 550.8 | 163.02 | 204.95 |
| 6 | 763.84 | 1155.40 | 175.77 | 198.75 | 703.08 | 747.30 | 492.59 | 832.10 | 232.19 | 267.65 | 117.18 | 166.95 | 416.64 | 601.55 | 223.51 | 272.95 | 145.39 | 188.15 |
| 7 | 552 | 730.62 | 68.08 | 76.26 | 193.2 | 252.15 | 331.2 | 441.57 | 190.44 | 241.08 | 57.04 | 83.64 | 218.96 | 399.75 | 170.2 | 191.88 | 92.92 | 131.61 |
| 8 | 743.54 | 663.00 | 65.54 | 84.66 | 39.55 | 85.68 | 412.45 | 298.86 | 257.64 | 252.96 | 257.64 | 210.12 | 135.60 | 119.34 | 158.20 | 154.02 | 75.71 | 92.82 |
